# Systematic Analysis of UFMylation Family Genes in Tissues of Mice with Metabolic Dysfunction-Associated Steatotic Liver Disease

**DOI:** 10.3390/genes16010031

**Published:** 2024-12-27

**Authors:** Mingdi Jiang, Chenlu Zhang, Zhengyao Zhang, Yingying Duan, Shuaiyong Qi, Qingyu Zeng, Jiabao Wang, Jiawen Zhang, Yu Jiang, Ying Wang, Yi Chen, Jiang Liu

**Affiliations:** 1 Zhejiang Key Laboratory of Medical Epigenetics, Department of Cell Biology and Genetics, School of Basic Medical Sciences, Hangzhou Normal University, Hangzhou 310036, China; jmd59527@163.com (M.J.); zcl19970930@126.com (C.Z.); duanyingying187@163.com (Y.D.); qishuaiyong@163.com (S.Q.); nhzengqingyu@163.com (Q.Z.); wang00jiabao@163.com (J.W.); jwzhangnu@163.com (J.Z.); 2 Zhejiang Key Laboratory of Medical Epigenetics, Department of Biochemistry and Molecular Biology, School of Basic Medical Sciences, Hangzhou Normal University, Hangzhou 310036, China; 15990114851@163.com (Z.Z.); flashingdancer@163.com (Y.W.); 3 Zhejiang Key Laboratory of Medical Epigenetics, Department of Human Anatomy and Histoembryology, School of Basic Medical Sciences, Hangzhou Normal University, Hangzhou 310036, China; 20220125@hznu.edu.cn

**Keywords:** obesity, MASLD, *ob/ob* mice, high-fat diet-fed mice, UFMylation

## Abstract

Background/Objectives: UFMylation, a newly identified ubiquitin-like modification, modulates a variety of physiological processes, including endoplasmic reticulum homeostasis maintenance, DNA damage response, embryonic development, and tumor progression. Recent reports showed that UFMylation plays a protective role in preventing liver steatosis and fibrosis, serving as a defender of liver homeostasis in the development of metabolic dysfunction-associated steatotic liver disease (MASLD). However, the regulation of UFMylation in MASLD remains unclear. This study aimed to determine the expressed patterns of UFMylation components in multiple tissues of leptin-deficient *ob/ob* mice and high-fat diet (HFD)-fed mice, which are mimicking the conditions of MASLD. Methods: The *ob/ob* mice and HFD-fed mice were sacrificed to collect tissues indicated in this study. Total RNA and proteins were extracted from tissues to examine the expressed patterns of UFMylation components, including UBA5, UFC1, UFL1, DDRGK1, UFSP1, UFSP2 and UFM1, by real-time PCR and western blot analysis. Results: The protein levels of UBA5, UFC1 and UFL1 were down-regulated in liver, brown adipose tissue (BAT) and inguinal white adipose tissue (iWAT), whereas the messenger RNA (mRNA) levels of *Ufl1* and *Ufsp1* were both decreased in skeletal muscle, BAT, iWAT and epididymal white adipose tissue (eWAT) of *ob/ob* mice. In contrast, the mRNA levels of *Ufsp1* in skeletal muscle, BAT, iWAT and heart, and the protein levels of UFL1 were decreased in BAT, iWAT, heart and cerebellum of HFD-fed mice. Conclusions: Our findings established the expressed profiles of UFMylaiton in multiple tissues of mice mimicking MASLD, indicating an important regulation for UFMylation in these tissues’ homeostasis maintenance.

## 1. Introduction

The global epidemic of obesity has been accompanied by a rising burden of metabolic dysfunction-associated steatotic liver disease (MASLD), formerly known as nonalcoholic fatty liver disease (NAFLD), which is a prevalent chronic liver disease worldwide that is characterized by hepatic steatosis, insulin resistance, dyslipidemia, and liver damage [1,2]. Both obesity and MASLD pathogenesis are multifactorial, and the genetic and environmental factors and metabolic factors are responsible for their development [2]. Accumulating evidence suggests that the dynamic post-translational modifications (PTMs) exerting diverse cellular outcomes also contribute to the pathogenesis of obesity and MASLD. For example, a very detailed portrait of the dynamic changes in protein phosphorylation was obtained in *ob/ob* mice, implicating that the kinase glycogen synthase kinase-3 (GSK3) is hyperactivated via phosphorylation, thereby regulating the insulin secretion in mice islets [3]. Ubiquitin was identified as a marker of cell injury in nonalcoholic steatohepatitis (NASH), which is now replaced with the term metabolic dysfunction-associated steatohepatitis (MASH) [1,4], and ubiquitylation also plays a vital role in the progression of MASLD via modulating different targets, such as tumor necrosis factor receptor-associated factor 6 (TRAF6), which can promote poly-ubiquitination and the subsequent activation of apoptosis signal-regulating kinase 1 (ASK1) in hepatocytes, thereby aggravating hepatic inflammation and fibrosis during MASH development [5]. Additionally, the global NEDDylated proteome in patients and mouse models with liver fibrosis demonstrated that NEDDylation inhibition downregulates the inflammatory response, consequently reducing cell damage and subsequent liver fibrosis [6]. Although much attention has focused on obesity and MASLD, the pathogenesis is not completely understood.

UFMylation, a newly identified ubiquitin-like modification, also requires a series of enzymatic cascades [7,8]. Mature ubiquitin-fold modifier 1 (UFM1) is generated from a UFM1 precursor cleaved by UFM1-specific eptidases 1 and 2 (UFSP1 and UFSP2) [9]. Subsequently, UFM1 is transferred to substrates via ubiquitin-like modifier-activating enzyme 5 (UBA5, E1), ubiquitin-fold modifier-conjugating enzyme 1 (UFC1, E2), and UFM1-specific ligase 1 (UFL1, E3) [7,8]. DDRGK domain-containing 1 (DDRGK1, also known as UFM1-binding protein 1 (UFBP1)) is associated with UFL1 to form an obligate heterodimer required for UFL1 E3 ligase activity [10]. To date, an array of UFMylation substrates are identified and well characterized, such as activating signal cointegrator 1 (ASC1) [10], meiotic recombination 11 homolog 1 (MRE11) [11], Histone H4 [12], p53 [13], retinoic acid-inducible gene 1 (RIG-1) [14], cytochrome b5 reductase 3 (CYB5R3) [15], and programmed death ligand-1 (PD-L1) [16], which led to a range of cellular processes and connected UFMylation to a variety of diseases, including hip dysplasia [17], kidney atrophy [18], heart failure [19], cancer, and neurodevelopmental disease [20,21,22,23,24]. Previous reports showed that UFMylation components are downregulated in MASH patients’ biopsies and disease mouse models [25,26]. Recently, a new study revealed that UFMylation of DDRGK1 can ameliorate obesity, hepatic lipogenesis, and insulin resistance in mice with MASLD, indicating an involvement of the UFMylation pathway in MASLD progression [27].

MASLD is a complex multisystem disease, and interactions between different organs contribute to MASLD pathogenesis and development [28]. To better understand the UFMylation pathway in MASLD regulatory networks, the messenger RNA (mRNA) and protein expression levels of UFMylation components were determined on more tissues of *ob/ob* mice and high-fat diet (HFD)-fed mice in this study. Our results showed that the expression levels of UFM1 and UFL1 are downregulated in the liver, skeletal muscle, brown adipose tissue (BAT), inguinal white adipose tissue (iWAT), epididymal white adipose tissue (eWAT), heart, and cerebrum of *ob/ob* mice, whereas the expression levels of UFL1 are decreased in BAT, iWAT, and the heart of HFD-fed mice. Together, our results suggest a new discovery for UFMylation in regulating the homeostasis of these metabolic organs, implying novel insights for understanding the pathophysiological mechanisms of metabolic diseases in mammals.

## 2. Materials and Methods

### 2.1. Antibodies

Antibodies used in this study were as follows: rabbit anti-UFL1 (HPA030559, Sigma, St. Louis, MO, USA), rabbit anti-DDRGK1 (HPA013373, Sigma), rabbit anti-UFM1 (ab109305, Abcam, Cambridge, MA, USA), rabbit anti-UFSP2 (ab185965, Abcam), rabbit anti-UFC1 (ab189252, Abcam), rabbit anti-UBA5 (ab177478, Abcam), rabbit anti-α-Tubulin (11224-1-AP, Proteintech, Chicago, IL, USA), mouse anti-GAPDH (M1310-2, HuaAn Biotechnology, Hangzhou, China), HRP-Goat Anti-Mouse IgG (A25012, Abbkine, Wuhan, China), and HRP-Mouse Anti-Rabbit IgG (A25022, Abbkine).

### 2.2. Animals

The *ob/ob* mice have been widely used as animal models in obesity and MASLD research, with the genetic background caused by leptin deficiency. For the analysis of the UFMylation family genes in *ob/ob* mice, five eight-week-old *ob/ob* male mice with the corresponding control C57BL/6J male mice were purchased from GemPharmatech (Nanjing, China). The C57BL/6J and *ob/ob* mice were allowed free access to water and a normal chow diet (NCD, 1010085, Synergy Biology, Jiangsu, China). The mice were sacrificed at ten weeks old, followed by the collection of tissue samples.

The HFD-fed mice mimic physiological conditions in humans and also are commonly used in obesity and MASLD research. For the analysis of the UFMylation family genes in HFD-fed mice, ten eight-week-old C57BL/6J male mice were purchased from GemPharmatech. The C57BL/6J male mice were randomized and separated into two groups matched by body weight, and the mice were fed an HFD (D12492, Research Diets, New Brunswick, NJ, USA) with 60% kcal or NCD for 8 weeks. The mice were sacrificed at sixteen weeks old, followed by the collection of tissue samples.

A comparison was performed in *ob/ob* mice vs. WT mice and HFD-fed mice vs. NCD-fed mice groups. The WT and NCD mice are different animals. All the mice were housed in an environment with a temperature of 22 ± 1 °C, a relative humidity of 50 ± 1%, and a light/dark cycle of 12/12 h. All animal studies (including the mouse euthanasia procedure) were approved and performed in compliance with the regulations and guidelines of the Animal Care and Use Committee of Hangzhou Normal University.

### 2.3. Western Blot Analysis

Tissues isolated from mice were lysed in a lysis buffer (150 mM NaCl, 50 mM Tris-HCl [pH 7.4], 1 mM EDTA, 1% NP40, and 0.1% SDS) with protease inhibitors. Whole protein samples were loaded and separated on a 12.5% (to determine UFC1 and UFM1) or 7.5% (to determine the rest of the proteins) sodium dodecyl sulfate–polyacrylamide gel electrophoresis (SDS-PAGE) followed by transferring the proteins onto polyvinylidene fluoride membranes. The membranes were blocked with a solution of 0.1% Tween 20/PBS (PBS-T) containing 5% fat-free milk (BS102, Biosharp, Hefei, China) for 1 h and were then incubated with primary antibodies overnight at 4 °C, followed by washing for 3 × 6 min with PBS-T buffer. The blots were re-blocked with PBS-T containing 5% fat-free milk, followed by incubation with HRP-Goat Anti-Mouse IgG or HRP-Mouse Anti-Rabbit IgG for 1 h at room temperature. After washing 3 × 6 min with PBS-T, the membranes were incubated with Western blot chemiluminescence reagents (FD8020, FDbio science, Hangzhou, China) for 1 min and then exposed to scanning with an automatic luminescence imaging system (5200, Tanon, Shanghai, China).

### 2.4. Real-Time PCR Assays

Total RNA was extracted from tissues using the Total RNApure Reagent (ZP401, Zoman Biotechnology, Beijing, China); complementary DNA was then prepared using the 5 × HiScript Q RT SuperMix (R122-01, Vazyme, Nanjing, China) for qPCR. Real-time PCR assays were performed using the AceQ qPCR SYBR Green Master Mix (Q111-02, Vazyme) with a CFX96 Real-time PCR Detection System (Bio-Rad, Los Angeles, CA, USA). The sequences of the primers used for real-time PCR are described in Table 1. The expression of UFMylation component mRNA in tissues was normalized to the corresponding *β-actin* mRNA. Student’s *t*-tests were applied to compare the two groups.

### 2.5. Statistical Analysis

All values are shown as mean standard deviations (SDs), and all experiments were repeated at least three times. Student’s *t*-tests were applied to compare the two groups. Differences with *p*-values < 0.05 were deemed statistically significant.

## 3. Results

**Figure 1:**
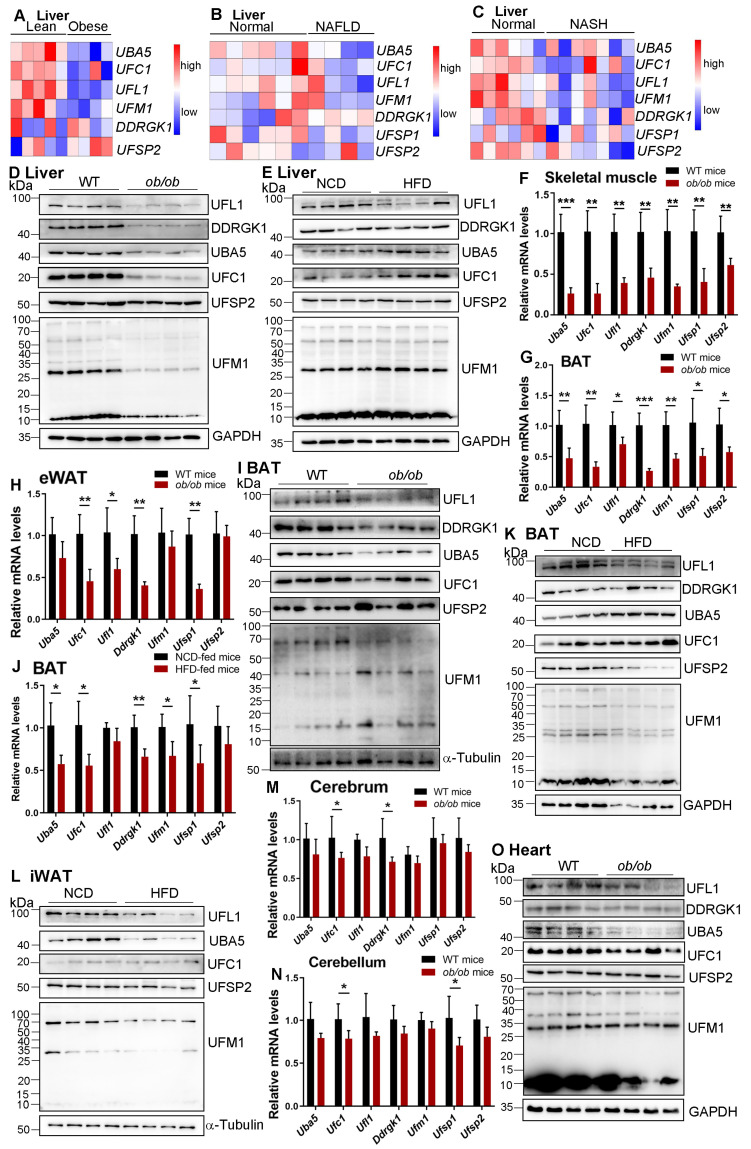
Systematic analysis of UFMylation components in the liver of patients and tissues of mice with MASLD. (**A**) The level of UFMylation components expression in the human liver with or without obesity in the Gene Expression Omnibus database (GSE15653). (**B**) The level of UFMylation components expression in the human liver with or without MASLD in the Gene Expression Omnibus database (GSE59045). (**C**) The level of UFMylation components expression in the human liver with or without MASH in the Gene Expression Omnibus database (GSE159676). (**D**) Liver samples were collected from wild-type and *ob/ob* mice and used to determine the expression of indicated proteins by a Western blot. *n* = 4. (**E**) Liver samples were collected from mice fed with NCD or HFD and used to determine the expression of indicated proteins by a Western blot. *n* = 4. Skeletal muscle (**F**), BAT (**G**), and eWAT (**H**) samples were collected from wild-type and *ob/ob* mice and used to determine the mRNA expression of indicated genes by real-time PCR. * *p* < 0.05; ** *p* < 0.01; *** *p* < 0.001 by Student’s *t*-test, *n* = 5. (**I**) BAT samples were collected from wild-type and *ob/ob* mice and used to determine the expression of indicated proteins by a Western blot. *n* = 4. (**J**) BAT samples were collected from mice fed with NCD or HFD and used to determine the mRNA expression of indicated genes by real-time PCR. * *p* < 0.05; ** *p* < 0.01 by Student’s *t*-test, *n* = 5. BAT (**K**) and iWAT (**L**) samples were collected from mice fed with NCD or HFD and used to determine the expression of indicated proteins by a Western blot. *n* = 4. Cerebrum (**M**) and cerebellum (**N**) samples were collected from wild-type and *ob/ob* mice and used to determine the mRNA expression of indicated genes by real-time PCR. * *p* < 0.05 by Student’s *t*-test, *n* = 5. (**O**) Heart samples were collected from wild-type and *ob/ob* mice and used to determine the expression of indicated proteins by a Western blot. *n* = 4.

Previous studies showed that the expression of UFM1 is substantially decreased in MASH and alcoholic hepatitis (AH) patients’ biopsies [25,26], indicating a fundamental role of UFMylation in liver homeostasis. As expected, the expression levels of hepatic *Uba5*, *Ufc1*, *Ufl1,* and *Ufm1* are significantly reduced in patients with obesity, MASLD, and MASH by analyzing the public data sets (Figure 1A–C). To further understand the regulation of UFMylation in MASLD progression, the mRNA and protein-expressed patterns of UFMylation components were determined by real-time PCR and Western blotting analysis in *ob/ob* mice and HFD-fed mice. Except for UFSP2, the protein expression level was unchanged, and our results showed that the protein expression levels of UBA5, UFC1, UFL1, UFM1, and DDRGK1 were significantly decreased in the livers of *ob/ob* mice compared with those of wild-type mice (Figure 1D), whereas there was only a minor decrease in the UFL1 protein level in the livers of HFD-fed mice compared with those of NCD-fed mice, accompanied with the unchanged expression levels of rest proteins (Figure 1E). Interestingly, there were no differences in *Uba5*, *Ufc1*, *Ufl1*, *Ufm1*, *Ddrgk1*, *Ufsp1*, and *Ufsp2* expression in the livers of *ob/ob* mice and HFD-fed mice compared with those of control mice (Figure S1A,B). Together, these observations indicated a context-dependent role of UFMylation in liver diseases, increasing the complexity of UFMylation regulation in liver homeostasis.

MASLD, as a common metabolic disease, is involved in multi-organ interaction [28]. Therefore, we examined the expression levels of UFMylation family genes in other metabolic organs in mice, including skeletal muscle, brown adipose tissue (BAT), inguinal white adipose tissue (iWAT), and epididymal white adipose tissue (eWAT). In *ob/ob* mice, the mRNA levels of all the detected UFMylation components in the skeletal muscle and BAT were dramatically decreased (Figure 1F,G). Similarly, the mRNA expression levels of *Ufc1*, *Ufl1*, *Ddrgk1,* and *Ufsp1* in eWAT, as well as *Ufl1* and *Ufsp1* in iWAT, were clearly reduced in *ob/ob* mice compared with those in wild-type mice (Figure 1H and Figure S2A). In addition, the protein expression levels of UBA5, UFC1, UFL1, and DDRGK1 in BAT, UBA5, UFC1, UFL1, and UFM1 in iWAT, and UFC1 and DDRGK1 in the skeletal muscle were significantly decreased in *ob/ob* mice (Figure 1I and Figure S2B,C). However, the protein expression levels of UFSP2 and UFM1 in BAT, DDRGK1 and UFSP2 in iWAT, and UFL1, UBA5, UFM1, and UFSP2 in the skeletal muscle were not significantly decreased in *ob/ob* mice (Figure 1I and Figure S2B,C). There were no significant differences in eWAT between *ob/ob* mice and wild-type mice in terms of the protein expression levels of UFMylation components (Figure S2D). Notably, the protein expression of UFSP1 was unable to be detected in all of these organs we described in this study, which is probably due to the bad quality of the antibody.

In HFD-fed mice, the mRNA expression level of *Ufsp1*, but not other UFMylation components, was decreased in the skeletal muscle and iWAT (Figure S3A,B), and the expression levels of all UFMylation family genes, except *Ufl1* and *Ddrgk1,* were reduced in BAT (Figure 1J). In contrast, no significant differences were detected in eWAT with regard to the expression of all UFMylation components between HFD-fed mice and NCD-fed mice (Figure S3C). Similar to our observation in *ob/ob* mice, the protein expression levels of UFL1, UFM1, and UFSP2 in BAT, as well as UBA5 and UFL1 in iWAT, were significantly decreased in HFD-fed mice (Figure 1K,L), whereas an increase in UFC1 expression was observed in eWAT in HFD-fed mice (Figure S3D). Furthermore, there were no differences in the UFMylation component expression in the skeletal muscle between HFD-fed mice and NCD-fed mice (Figure S3E). Together, our results revealed that UFMylation components play an essential role in skeletal muscle and adipose tissue homeostasis, contributing to MASLD development.

We further characterized the UFMylation-expressed patterns in the heart, cerebrum, and cerebellum in mouse models, and our results showed that the mRNA expression levels of all UFMylation family genes were not changed in the heart of *ob/ob* mice (Figure S4A). Additionally, the expression levels of *Ufc1* and *Ddrgk1* in the cerebrum and *Ufc1* and *Ufsp1* in the cerebellum were obviously downregulated in *ob/ob* mice compared to those of wild-type mice. However, the expression levels of the rest genes in the cerebrum or cerebellum were unchanged (Figure 1M,N). In contrast to the observations in the heart, we found that the protein expression levels of UBA5, UFC1, UFL1, and UFM1 were significantly decreased in *ob/ob* mice (Figure 1O). The protein expression levels of UFC1, UFL1, DDRGK1, and UFSP2 were also reduced in the cerebrum of *ob/ob* mice (Figure S4B). Interestingly, the UFC1 protein expression level was clearly upregulated in the cerebellum of *ob/ob* mice compared with those of control mice (Figure S4C), indicating that UFC1 might exert an independent role of UFMylation in brain homeostasis.

Next, we examined the expression levels of UFMylation components in the heart, cerebrum, and cerebellum in HFD-fed mice. Our results showed that the expression levels of *Uba5*, *Ufl1*, *Ddrgk1*, *Ufsp1,* and *Ufsp2* in the heart were decreased compared to NCD-fed mice (Figure S5A). In the cerebrum and cerebellum, our findings did not show any significant expression differences of these UFMylation family genes between the NCD-fed mice and the HFD-fed mice (Figure S5B,C). Furthermore, the protein expression levels of UBA5, UFL1, and DDRGK1 were reduced in the heart of HFD-fed mice, accompanied by the unchanged expression levels of UFSP2, UFC1, and UFM1 proteins (Figure S5D). In the cerebrum, we did not find any differences between the NCD-fed mice and the HFD-fed mice with regard to the protein expression levels of UFMylation components (Figure S5E). In the cerebellum, the protein expression levels of UFL1 and DDRGK1 were observed with a minor decrease in HFD-fed mice compared to NCD-fed mice (Figure S5F). Collectively, these expressed patterns suggest potentially essential roles of UFMylation in heart and brain function.

## 4. Conclusions

MASLD is a complex disease that is involved with multi-organ interaction and modulated by numerous mechanisms in its pathogenesis. Although many features of MASLD pathogenesis are known, the regulation of post-translational modifications in MASLD is not well delineated. UFMylation is a newly identified ubiquitin-like modification, whose biological significance is largely unknown. In this study, we carried out a systematic analysis of UFMylation family gene expression in multiple tissues of the mice, mimicking MASLD, and our results reveal interesting findings that imply novel physiological functions for UFMylation in different organs in MASLD development and progression.

In the present study, we found that the expression of UFMylation components, especially UFL1, was decreased in the livers of *ob/ob* mice and HFD-fed mice at the protein levels but not the mRNA levels, indicating that UFL1 should be regulated post-translationally in mice livers with MASLD conditions. Interestingly, UFL1 can be phosphorylated by AMP-activated protein kinase (AMPK), which leads to the reduction in PD-1 UFMylation and the subsequent destabilization of PD-1, consequently enhancing CD8^+^ T cell activation and anti-tumor immunity. By contrast, the inhibition of AMPK activity elevated PD-1 UFMylation and stabilization to promote tumor growth [29]. Considering that AMPK negatively regulates mTOR signaling [30], it is not surprising that the hepatocyte-specific deletion of *Ufl1* or *Ddrgk1* in mice results in liver injury and increases susceptibility to HFD-induced fatty liver and diethylnitrosamine (DEN)-induced hepatocellular carcinoma by the activation of mTOR signaling [31]. Another interesting observation is that UFMylation components are involved in skeletal muscle and adipose tissue homeostasis in most cases of *ob/ob* mice and HFD-fed mice, indicating a comprehensive regulation of UFMylation and a complex crosstalk between these metabolic tissues, which may contribute to MASLD progression. We also found a significant decrease in UFL1 in the heart of both mouse models, which is consistent with a previous report that Ufl1 is downregulated in human failing hearts, and it can protect against heart failure [19]. Taken together, it is conceivable to conclude that UFMylation functions as a defender of multiple organ homeostasis maintenance, and disruption of the homeostasis may exacerbate MASLD.

Accumulating evidence showed that the UFMylation system is implicated in numerous cellular functions and human diseases [32], indicating that components of the UFMylation system are potential therapeutic targets. To date, several inhibitors of UFMylation have been discovered. Usenamine A, aUBA5-inhibiting compound, can effectively inhibit breast cancer cell proliferation and invasion [33]. Compound-8, a covalent inhibitor of UFSP2, has also been found to promote UFMylation activity and contribute to anti-PD-1 immunotherapy [16]. Therefore, chemicals targeting UFMylation present novel and promising therapeutic strategies in the treatment of MASLD and other metabolic diseases.

### Limitations of This Study

Despite establishing the expressed profiles of UFMylaiton in multiple organs of mice mimicking MASLD, the mechanisms and functions of UFMylation in these organs’ homeostasis and their crosstalk remain unclear, largely because of the elusive identifications of bona fide UFMylation substrates in regulating these organs homeostasis. Moreover, most of the research articles in the literature about UFMylation research are focused on illustrating the substrates for UFMylation and their biological functions, whereas the PTMs of UFMylation family proteins are rarely reported, limiting our understanding of the PTMs’ regulation of themselves. Therefore, it will be important to clarify the regulation of UFMylation components themselves, as well as the regulation and functions of their targets. Nevertheless, our findings provide novel information that will guide multiple research directions to add to our knowledge concerning the physiological function of UFMylation in MASLD and other metabolic diseases.

## Figures and Tables

**Table 1:** Primer sequences used in real-time PCR.

Gene (Mus Musculus)	Primer Sequence (5′-3′)
*Ufl1-Forward*	CTGGGACAACTGATTGATGAGAA
*Ufl1-Reverse*	AGGAAGGTCATAGGCTTTACACA
*Ufm1-Forward*	GCTGCCGTACAAAGTTCTCAG
*Ufm1-Reverse*	GTGCTTCAGGAAAACATTCCCA
*Uba5-Forward*	GAGATGAGCGACGAGGTGTTG
*Uba5-Reverse*	ACAGCGTAGGTACGGATTTTCT
*Ufc1-Forward*	CGGGTCGTGTCTGAGATCC
*Ufc1-Reverse*	GGTCCCTTCCTTGTTGGACT
*Ddrgk1-Forward*	CCCTGGGTGTATCTGGTGG
*Ddrgk1-Reverse*	CATTGTGCAGTGGTTCTCCGT
*Ufsp1-Forward*	ATCACTATGGTTGCGATGGACT
*Ufsp1-Reverse*	GCCGATCCAGTTACGGGAG
*Ufsp2-Forward*	TATCAAGAACGCACTGCGACA
*Ufsp2-Reverse*	CCACAGGTACACGGAACTGTT
*β-actin-Forward*	CTCAGGAGGAGCAATGATCTTGAT
*β-actin-Reverse*	TACCACCATGTACCCAGGCA

## Data Availability

All relevant data are included within the article and its Supplementary Information files or from the corresponding authors on request.

## Supplementary Materials

The following supporting information can be downloaded at www.mdpi.com/article/10.3390/genes16010031/s1, Figure S1: The mRNA expression levels of UFMylation components in liver of mice with MASLD; Figure S2: The levels of UFMylation components in iWAT, skeletal muscle and eWAT of *ob/ob* mice; Figure S3: The levels of UFMylation components in skeletal muscle, iWAT and eWAT of HFD-fed mice; Figure S4: The levels of UFMylation components in heart, cerebrum and cerebellum of *ob/ob* mice; Figure S5: The levels of UFMylation components in heart, cerebrum and cerebellum of HFD-fed mice.
