# Supplementary material for: Systematic Analysis of UFMylation Family Genes in Tissues of Mice with Metabolic Dysfunction-Associated Steatotic Liver Disease
Source: Genes (Basel). 2024 Dec 27;16(1):31. doi: 10.3390/genes16010031 (PMC11765366; doi:10.3390/genes16010031)
Supplement: Supplementary file 1 [file genes-16-00031-s001.zip › genes-3388134-supplementary.pdf]

Systematic analysis of UFMylation family genes in tissues of mice with metabolic dysfunction-associated steatotic liver disease

Figure S1

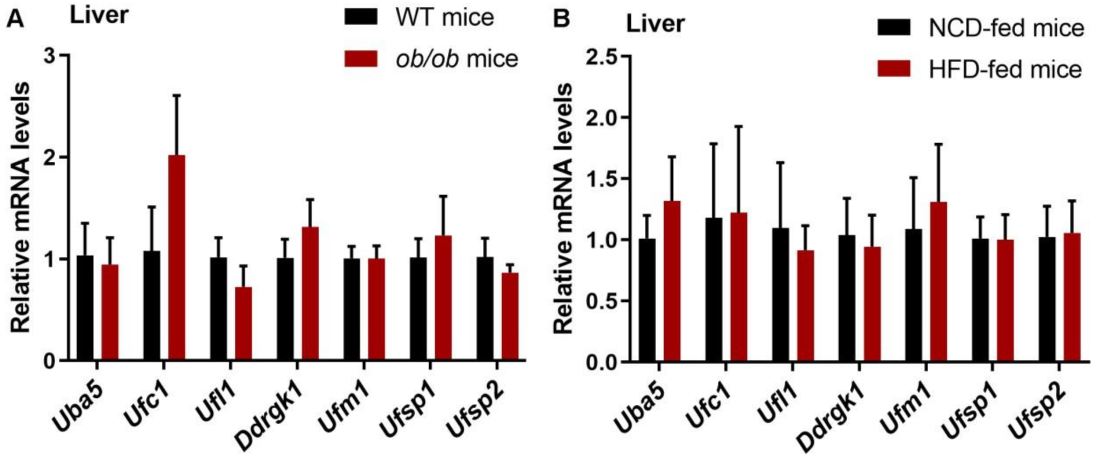

Figure S1. The mRNA expression levels of UFMylation components in liver of mice with MASLD. (**A**) Liver samples were collected from wild-type and *ob*/*ob* mice, and used to determine the mRNA expression of indicated genes by real-time PCR. *n* = 5. (**B**) Liver samples were collected from mice fed with NCD or HFD, and used to determine the mRNA expression of indicated genes by real-time PCR. *n* = 5.

Figure S2

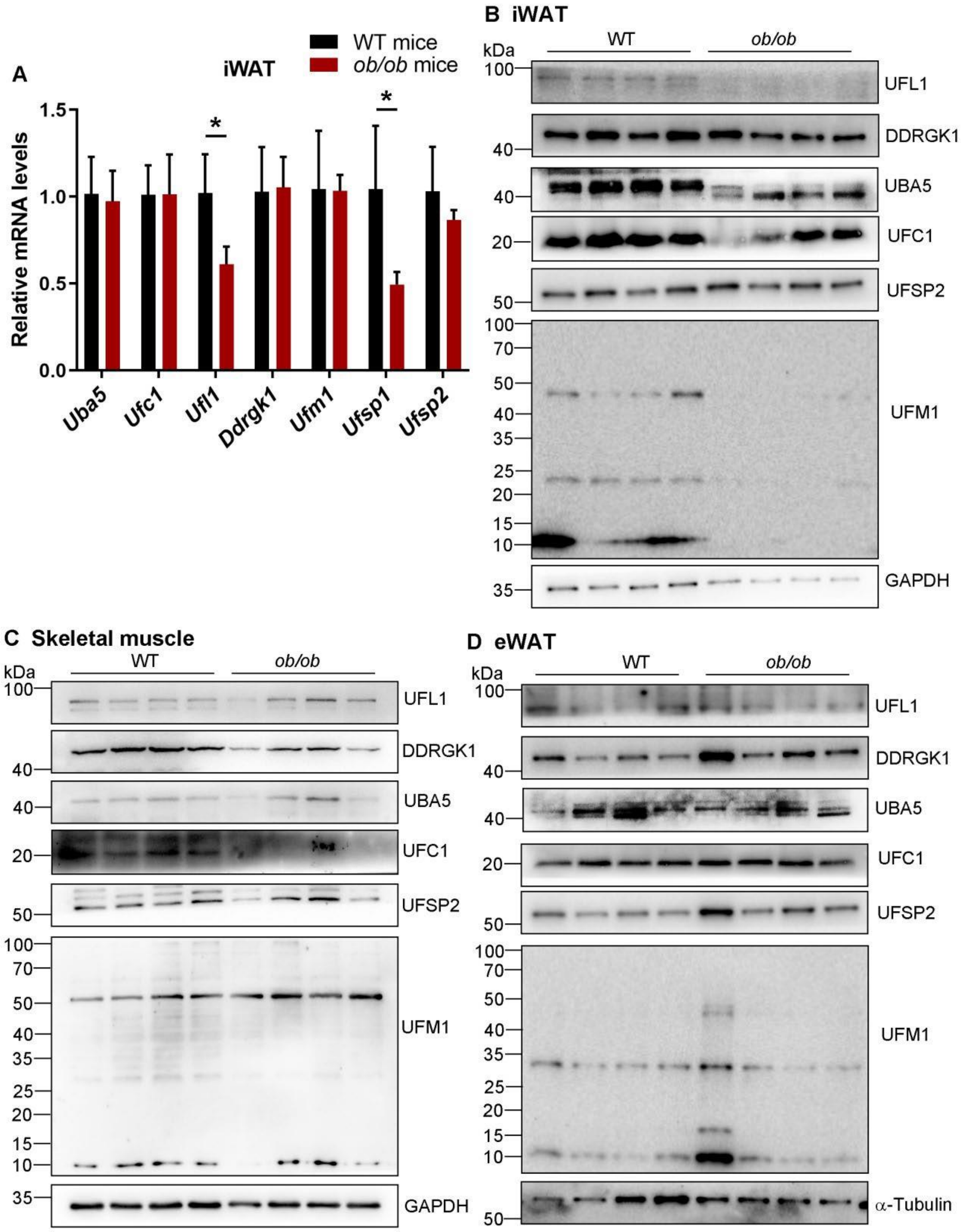

Figure S2. The levels of UFMylation components in iWAT, skeletal muscle and eWAT of *ob/ob* mice. (**A**) iWAT samples were collected from WT and *ob/ob* mice, and used to determine the mRNA expression of indicated genes by real-time PCR. * $p < 0.05$ by Student's *t*-test, $n = 5$. iWAT (**B**), Skeletal muscle (**C**) and eWAT (**D**) samples were collected from wild-type and *ob/ob* mice, and used to determine the expression of indicated proteins by Western blot. $n = 4$.

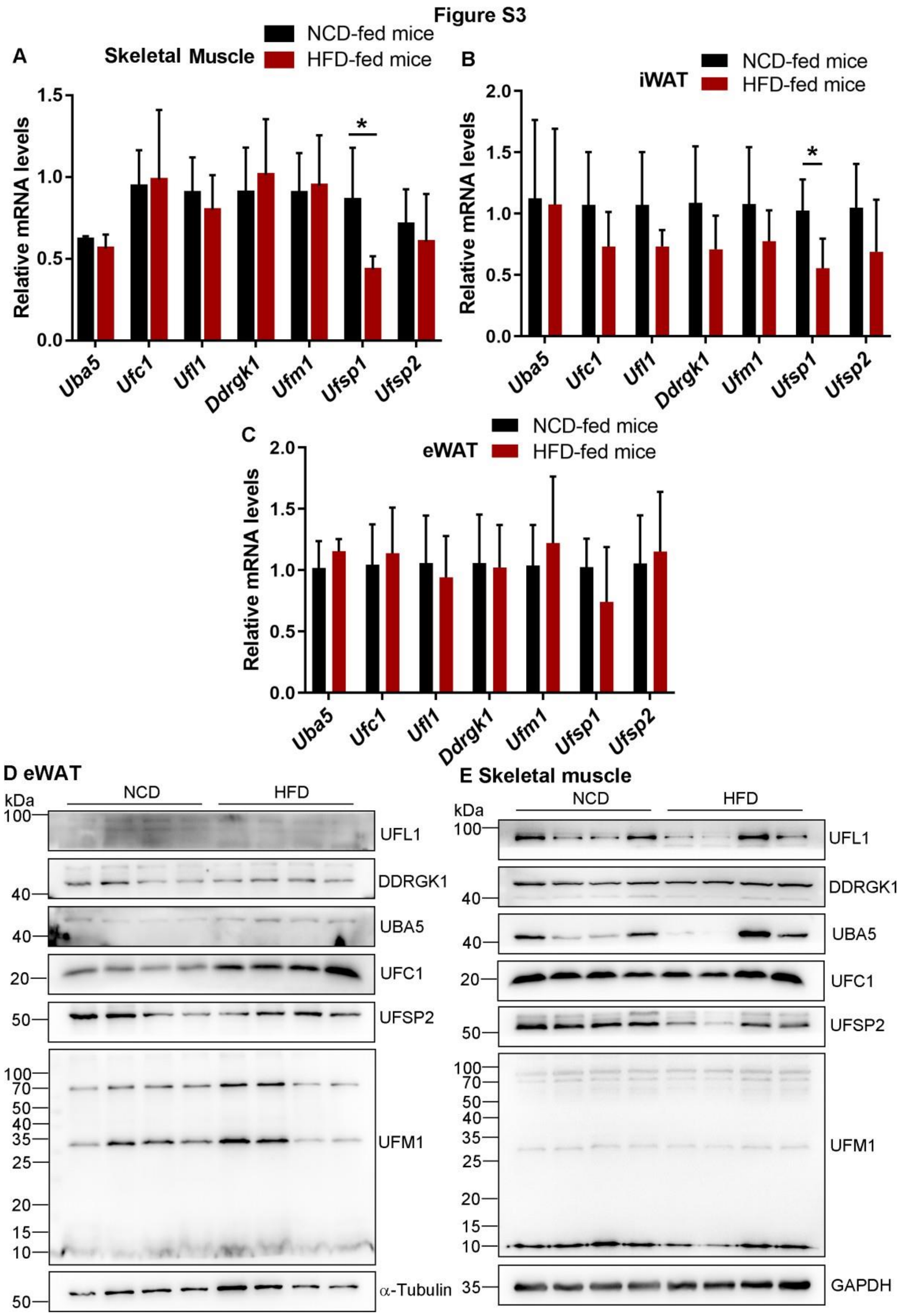

Figure S3. The levels of UFMylation components in skeletal muscle, iWAT and eWAT of HFD-fed mice. Skeletal muscle (**A**), iWAT (**B**) and eWAT (**C**) samples were collected from mice fed with NCD or HFD, and used to determine the mRNA expression of indicated genes by real-time PCR. * $p < 0.05$ by Student's t-test, $n = 5$. eWAT (**D**) and skeletal muscle (**E**) and samples were collected from mice fed with NCD or HFD, and used to determine the expression of indicated proteins by Western blot. $n = 4$.

Figure S4

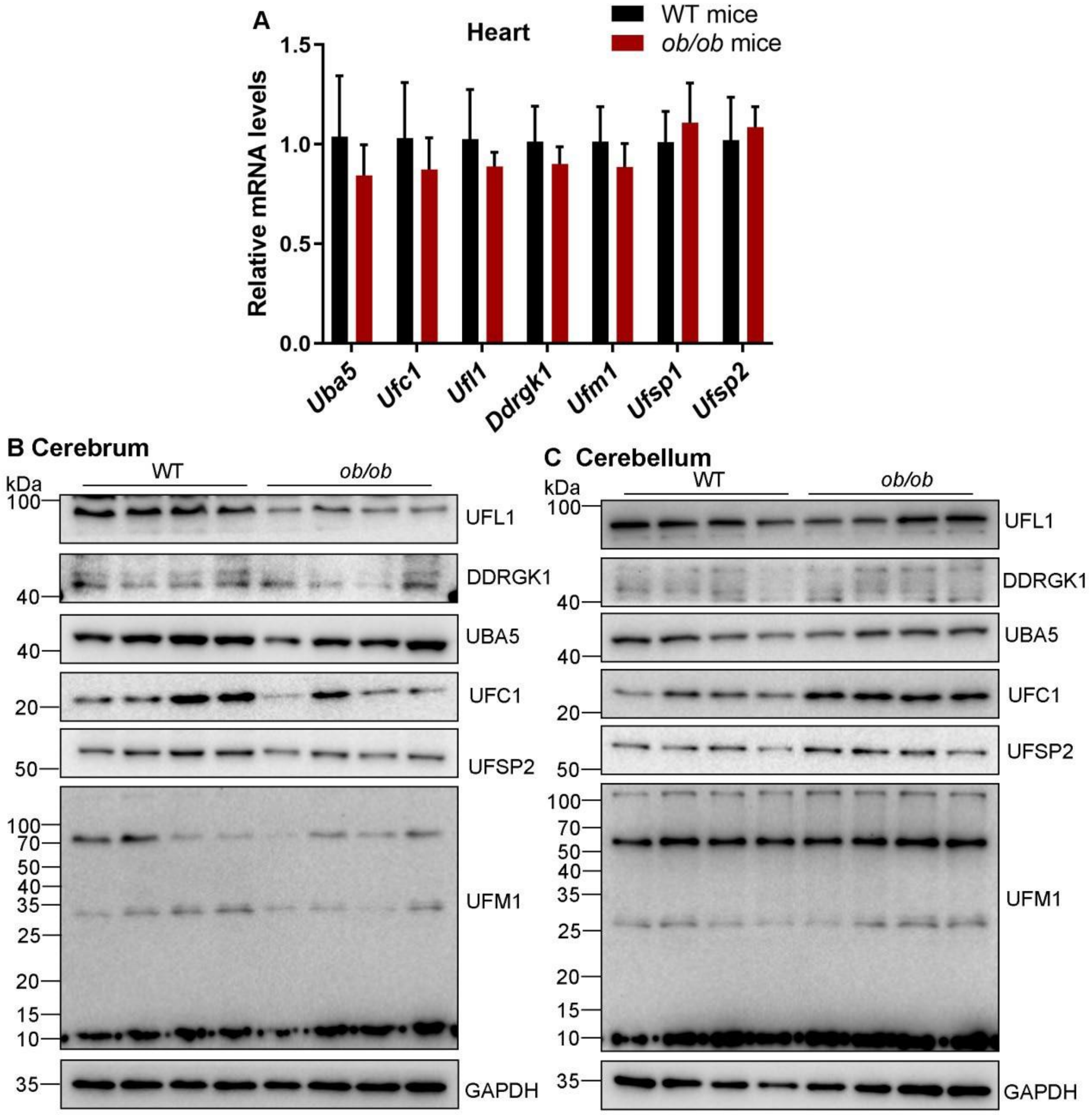

Figure S4. The levels of UFMylation components in heart, cerebrum and cerebellum of *ob*/*ob* mice. (**A**) Heart samples were collected from wild-type and *ob*/*ob* mice, and used to determine the mRNA expression of indicated genes by real-time PCR. *n* = 5. Cerebrum (**B**) and cerebellum (**C**) samples were collected from wild-type and *ob*/*ob* mice, and used to determine the expression of indicated proteins by Western blot. *n* = 4.

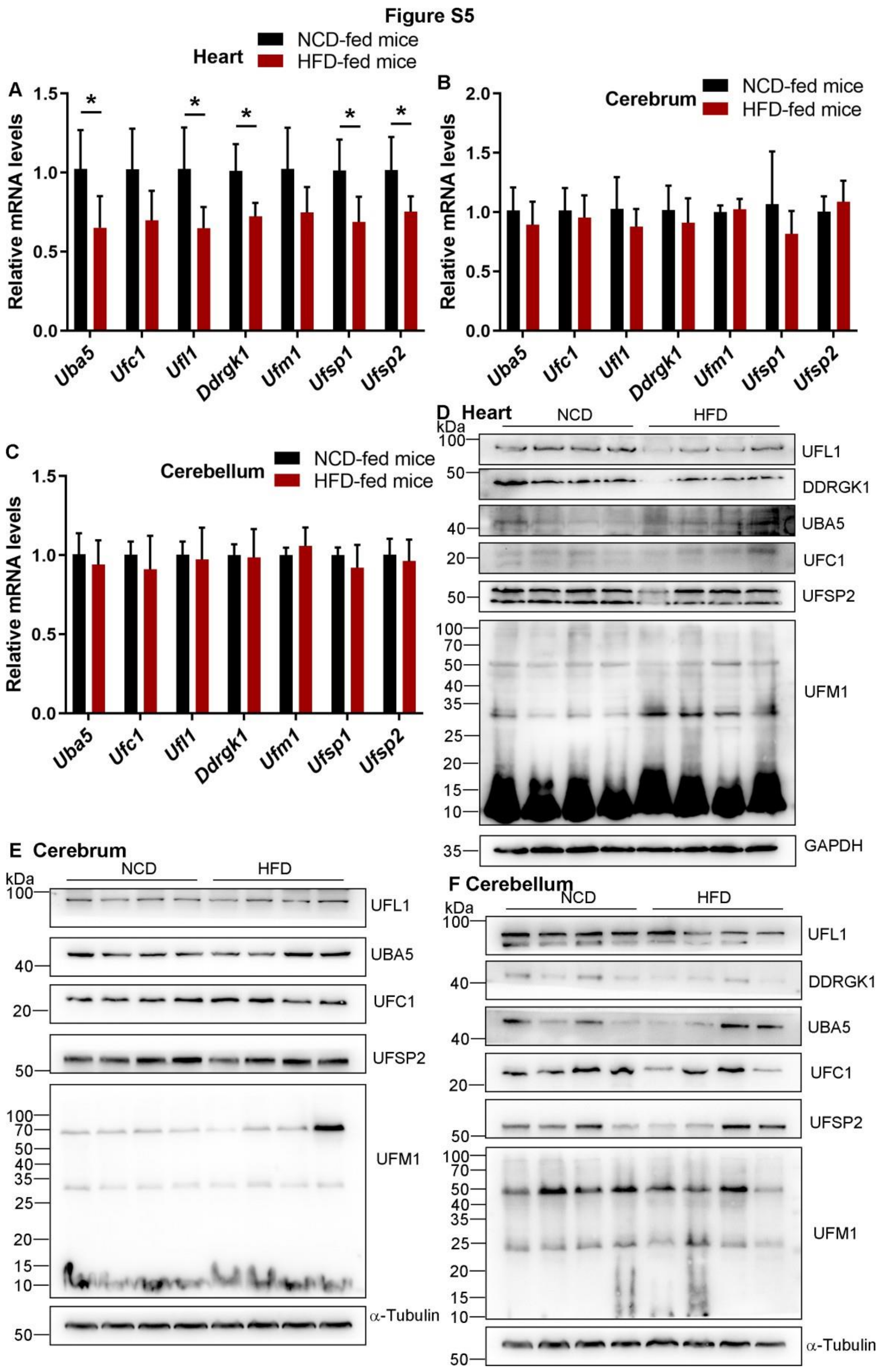

Figure S5. The levels of UFMylation components in heart, cerebrum and cerebellum of HFD-fed mice. Heart (**A**), cerebrum (**B**) and cerebellum (**C**) samples were collected from mice fed with NCD or HFD, and used to determine the mRNA expression of indicated genes by real-time PCR. * $p < 0.05$ by Student's *t*-test, $n = 5$. Heart (**D**), cerebrum (**E**) and cerebellum (**F**) samples were collected from mice fed with NCD or HFD, and used to determine the expression of indicated proteins by Western blot. $n = 4$.
